# IMPACT AND HEALTHCARE UTILIZATION PATTERNS OF AN ORTHOPEDIC TELEMEDICINE PROGRAM IN BRAZIL

**DOI:** 10.1590/1413-785220253302e292544

**Published:** 2025-10-13

**Authors:** FABIO SEIJI MAZZI YAMAGUCHI, VITOR MATHEUS SILVA, HECTOR FUGIHARA KROES, JORGE DOS SANTOS SILVA, RAPHAEL MARTUS MARCON, HENRIQUE MELO DE CAMPOS GURGEL

**Affiliations:** 1. Universidade de Sao Paulo, Faculdade de Medicina, Hospital das Clinicas, Instituto de Ortopedia e Traumatologia (HCFMUSP), Sao Paulo, SP, Brazil.; 2. Universidade de Sao Paulo, Faculdade de Medicina (FMUSP), Sao Paulo, SP, Brazil.

**Keywords:** Telemedicine, Social Vulnerability, Orthopedics, Telemedicina, Vulnerabilidade Social, Ortopedia

## Abstract

**Objective:**

To examine the relationship between place of residence, social vulnerability, and telemedicine access among orthopedic patients at a tertiary hospital in São Paulo, Brazil.

**Methods:**

A cross-sectional, comparative study analyzing sociodemographic, economic, and geographic characteristics of 475 patients who attended telemedicine consultations between August 2022 and August 2023. Patients were grouped by their Social Vulnerability Index (IVS) to assess differences in travel distances, costs, and accessibility challenges. The analysis included comparisons of demographic factors, financial burdens, and telemedicine’s impact on reducing travel constraints.

**Results:**

Telemedicine consultations avoided an average travel distance of 211 km and saved approximately 44 USD per consultation. Patients from higher IVS regions had significantly greater travel distances, longer travel times, lower formal education levels, and were more likely to be younger, male, and of non-White race. Additionally, these patients more frequently reported missing work for in-person visits. Telemedicine access disparities reflect broader socioeconomic and geographic inequalities.

**Conclusions:**

This study highlights how spatial and socioeconomic vulnerabilities shape telemedicine access in Brazil. Travel burdens and job insecurity disproportionately affect vulnerable groups, while social support mitigates barriers for older adults. Level of Evidence III; Cross-Sectional Study.

## RESUMO

**Objetivo:**

Examinar a relação entre local de residência, vulnerabilidade social e acesso à telemedicina entre pacientes ortopédicos de um hospital terciário em São Paulo, Brasil.

**Métodos:**

Estudo transversal e comparativo que analisou características sociodemográficas, econômicas e geográficas de 475 pacientes que participaram de consultas por telemedicina entre agosto de 2022 e agosto de 2023. Os pacientes foram agrupados conforme seu Índice de Vulnerabilidade Social (IVS) para avaliar diferenças em distâncias percorridas, custos e desafios de acessibilidade. A análise incluiu comparações de fatores demográficos, encargos financeiros e o impacto da telemedicina na redução de restrições ao deslocamento.

**Resultados:**

As consultas por telemedicina evitaram, em média, deslocamento de 211 km e economizaram aproximadamente 44 dólares por consulta. Pacientes de regiões com maior IVS apresentaram distâncias significativamente maiores, tempos de viagem mais longos, menor escolaridade e eram, com mais frequência, mais jovens, do sexo masculino e de raça não branca. Além disso, relataram mais frequentemente faltar ao trabalho para consultas presenciais.

**Conclusões:**

Este estudo destaca como vulnerabilidades espaciais e socioeconômicas moldam o acesso à telemedicina no Brasil. O ônus do deslocamento e a insegurança no trabalho afetam desproporcionalmente os grupos vulneráveis, enquanto o apoio social contribui para mitigar barreiras entre os idosos. Nível de Evidência III; Estudo Transversal.

## INTRODUCTION

Brazil’s vast geography and regional disparities create significant barriers to equitable healthcare access.^1^ Even in urban centers like São Paulo, socioeconomic inequalities lead to uneven healthcare distribution, with low-income communities experiencing greater challenges in accessing specialized care.^2^

Telemedicine has emerged as a promising solution to bridge gaps by reducing geographic and economic barriers to care, a process that was significantly accelerated by the COVID-19 pandemic. Telemedicine minimizes the need for costly and time-consuming trips to healthcare facilities, improves access for isolated communities, and ensures continuity of care by allowing patients to receive medical consultations remotely.^3^ For patients living in remote regions or underserved urban areas, telemedicine can represent not only a lifeline for timely medical care but also significant economic relief by reducing travel-related expenses and the opportunity costs associated with missing work or other responsibilities.^4-5^ The widespread use of mobile devices, such as smartphones, enhances the feasibility of expanding this modality, even among vulnerable populations.^6-9^

Among orthopedic patients, these benefits are especially significant, as their conditions often involve mobility limitations, chronic pain, or the need for frequent post-surgical follow-ups that require long-term management.^10^ For individuals with physical disabilities or those recovering from procedures, traveling to healthcare facilities can be particularly burdensome, increasing the risk of delays or interruptions in care.^10^

By evaluating the economic and social impacts of telemedicine and their relationship to socioeconomic conditions, this study seeks to highlight its role as a tool for reducing healthcare disparities in Brazil. The findings will provide a better understanding of how geographic distance, financial burdens, and social inequities influence patients’ access to care, helping to guide policies aimed at improving healthcare delivery for the most vulnerable populations. In a country as geographically and socially diverse as Brazil, such knowledge is essential for addressing regional inequities and ensuring that telemedicine fulfills its promise of expanding access to quality healthcare for all.

## METHODS

This single-center, cross-sectional observational study assessed the perceptions of telemedicine patients between August 2022 and August 2023 using questionnaires. Ethical approval was obtained from the Research Ethics Committee of the Hospital das Clínicas, Faculty of Medicine, University of São Paulo (approval number 5.022.929; CAAE: 51877521.0.0000.0068). Patients provided consent through a digital informed consent form (ICF) and answered a questionnaire providing information on their demographic characteristics, orthopedic care at our facility, and travel plans for a hypothetical in-person consultation at our hospital. Social vulnerability was indirectly measured.

### Population

The study included patients who attended at least one telemedicine outpatient consultation, provided by various orthopedic subspecialties, between August 2022 and August 2023 at a single, orthopaedics specialized, quaternary care hospital in Brazil. Exclusion criteria included individuals who did not sign the informed consent form or who withdrew from the study at any stage.

### Social Vulnerability

Social vulnerability was indirectly assessed through patients’ place of residence. Addresses obtained from hospital records were matched with a publicly available IVS, developed by the governmental research institute IPEA.^11^ The IVS is a composite measure that evaluates social and economic vulnerability in a given region, incorporating indicators such as income, education, housing conditions, employment rates, and access to essential services like healthcare and sanitation. To ensure greater specificity, we prioritized IVS data based on Human Development Units (UDHs), the most detailed geographic level available. When UDH-level data were unavailable, IVS values at the municipal (city) level were used.

### Distance and Travel Time

The coordinates of the hospital and patients’ residences were manually obtained via Google Maps using ‘Clipcoords v0.1.0’ software for assistance.^12^ Travel distances were calculated using a custom Python-based algorithm that employed Google’s geocoding API via Google Cloud.^13^ Travel times were manually determined by inputting the coordinates into the “Google Maps” website, selecting 8 a.m. on a Monday as the arrival time and following the first suggested route. The “car” option was chosen for individuals traveling by private cars or publicly funded vans/ambulances, while the “public transport” option was selected for those using mass transit public transportation.

### Travel Cost

To assess potential transport cost savings from telemedicine consultations, we used a hybrid method combining self-reported and indirect cost measures based on the mode of transport. This approach accounted for the complexity of estimating public transportation costs in São Paulo, which can vary significantly depending on patient access to government-subsidized discounts and integrated fare systems that lower expenses for users relying on multiple transport lines. In contrast, private transportation costs could be estimated more directly using an indirect measure of fuel expenses.

For individuals using a private car, travel costs were based on fuel consumption, which was estimated considering twice the travel distance previously obtained, the fuel efficiency of ten kilometers per liter, and BRL 6.12 (six Brazilian reais and 12 cents) per liter as the price of gasoline.^14,15^ For public transportation, costs were informed by patients through the questionnaire. A rate of 2.3 BRL (two Brazilian reais and 30 cents) per USD was used to convert costs to international standards and facilitate comparison as per the “CCEMG–EPPI Centre Cost Converter”.^16^

### Statistical Analysis

The Wilcoxon rank-sum test with continuity correction was applied to compare continuous variables between two independent groups. When comparing continuous variables across more than two independent groups, the Kruskal-Wallis test was employed. Correlations between continuous variables were assessed using Spearman’s rank correlation coefficient. Categorical variables were analyzed using Pearson’s chi-squared test. To account for potential confounding effects, partial correlations were performed. Statistical significance was set at p < 0.05, and all analyses were conducted using R version 4.3.1. (RStudio v2023.09.1).

## RESULTS

We collected data from 475 eligible participants, comprising 206 men (43%) and 269 women (57%). The participants had an average age of 53 years (SD = 16 years) and were categorized into three groups: 18 young adults, 296 adults, and 161 elderly individuals (>60 years). Regarding education, 22% had completed higher education, 7.8% had incomplete higher education, 29% had completed high school, 6.1% had incomplete high school, 9.7% had completed elementary school, 24% had incomplete elementary school, and 2.5% had no formal education. Most participants identified as white (49%) or mixed race (38%), while 11% identified as Black, 1.1% as Asian, and 0.6% as Indigenous (Table 1).

**Table 1:** Cohort’s sociodemographic data.

Characteristic	N = 475
**Age (years)**	
Mean (SD)	53 (16)
Median (IQR)	54 (43, 65)
Range	4, 91
**Age groups**	
Children	18 (3.8%)
Adults	296 (62%)
Elderly	161 (34%)
**Sex**	
Men	206 (43%)
Women	269 (57%)
**Race**	
White	235 (49%)
Mixed race	179 (38%)
Black	53 (11%)
Asian	5 (1.1%)
Indigenous	3 (0.6%)
**Education**	
No formal education	12 (2.5%)
Incomplete elementary school	112 (24%)
Completed elementary school	46 (9.7%)
Incomplete high school	29 (6.1%)
Completed high school	136 (29%)
Incomplete higher education	37 (7.8%)
Completed higher education	103 (22%)

**Figure 1:**
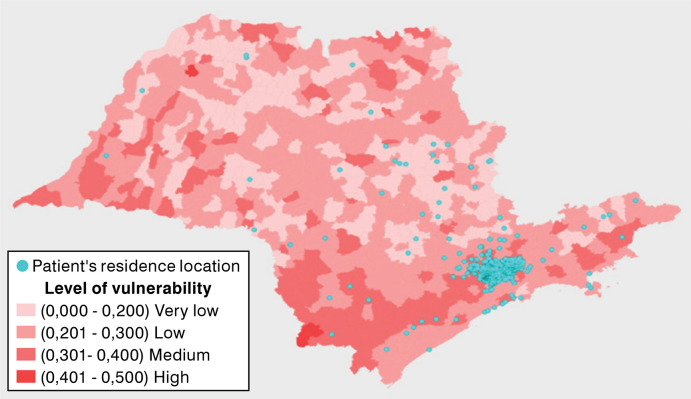
Map of the State of São Paulo highlighting cities, their respective vulnerability levels, and the place of residence of each patient.

**Figure 2:**
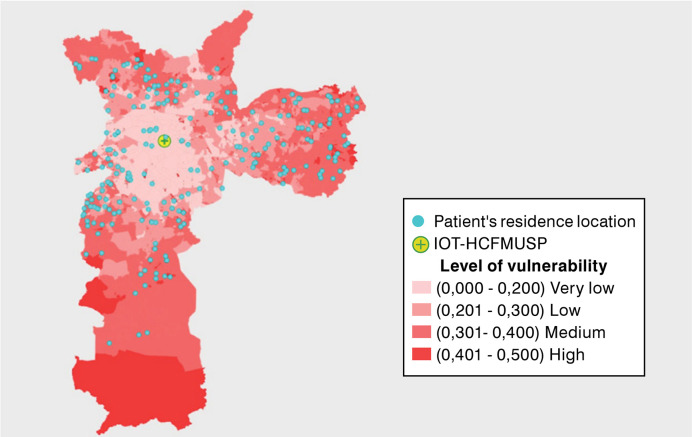
Map of the City of São Paulo highlighting Human Development Units, their respective vulnerability levels, and the place of residence of each patient.

Most patients resided in the hospital’s city or nearby areas, with 231 from the city of São Paulo, 221 from other cities in the state, and 23 from outside the state (Figures 1 and 2). The median travel distance avoided through telemedicine was 54 km (IQR 34-96 km), averaging a distance of 211 km. The median travel time to an in-person appointment was 170 minutes (IQR 130-222 min), resulting in an average travel time avoided of 286 minutes. Travel distance and time avoided were much more relevant in groups from outside the city of São Paulo (Table 2). Among the participants, 343 (72%) would have relied on public transportation, of whom 140 (28%) had access to free fare. Additionally, 229 (49%) of patients would have been accompanied to the appointment. Regarding teleconsultations, 86% used a mobile phone, 11% used a computer, and 3.2% reported using both devices. Telemedicine resulted in an average cost saving of 44 USD per patient.

**Table 2:** Telemedicine program socioeconomic impact and utilization patterns according to origin.

Characteristic	City of São Paulo N = 231	Another City in the State N = 221	Out of State N = 23	Total N = 475
**Round-trip distance saved (km)**				
Mean (SD)	39 (17)	185 (224)	2,184 (1,972)	211 (639)
Median (IQR)	36 (26, 50)	84 (54, 222)	1,274 (852, 2,867)	54 (34, 96)
Range	2, 92	22, 1,250	318, 7,758	2, 7,758
Sum	9,016	40,838	50,240	100,094
**Round-trip time saved (min)**				
Mean (SD)	138 (47)	292 (261)	1,716 (1,620)	286 (514)
Median (IQR)	140 (109, 170)	200 (160, 320)	1,000 (610, 2,360)	170 (130, 222)
Range	0, 274	12, 2,320	360, 6,600	0, 6,600
Sum	31,956	64,546	39,460	135,962
**Travel Expenses avoided (USD)**				
Mean (SD)	9 (11)	31 (52)	528 (512)	44 (160)
Median (IQR)	8 (0, 15)	17 (0, 28)	304 (200, 891)	11 (0, 19)
Range	0, 122	0, 277	0, 2,064	0, 2,064
Sum	2,166	6,822	12,135	21,122
**Utilized device**				
Only cellphone	194 (84%)	198 (90%)	18 (78%)	410 (86%)
Only computer	26 (11%)	20 (9.0%)	4 (17%)	50 (11%)
Cellphone or computer	11 (4.8%)	3 (1.4%)	1 (4.3%)	15 (3.2%)
Public transportation usage	169 (73%)	161 (73%)	13 (57%)	343 (72%)
Transportation gratuity	71 (31%)	67 (30%)	2 (8.7%)	140 (29%)
Accompanying person	117 (51%)	104 (47%)	8 (35%)	229 (48%)
Patient work absence	61 (26%)	70 (32%)	9 (39%)	140 (29%)
Accompanying person work absence	64 (28%)	78 (35%)	10 (43%)	152 (32%)

**Figure 3:**
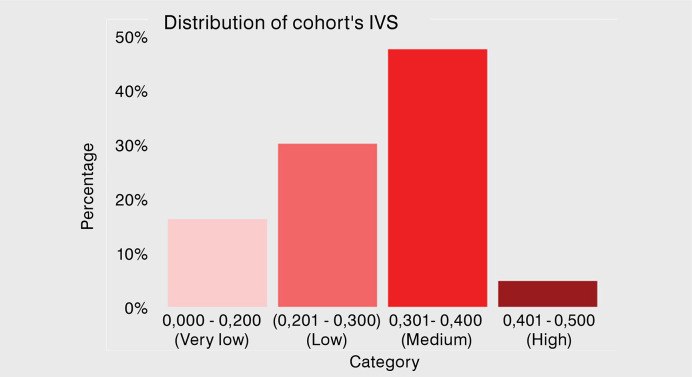
Vertical bar chart illustrating the distribution of the cohort's Social Vulnerability Index (IVS) categories. The graph shows the proportion of patients in each vulnerability range, with the majority falling within the Medium (0.301-0.400) and Low (0.201-0.300) categories, while fewer individuals are in the Very Low (0.000-0.200) and High (0.401-0.500) categories.

**Figure 4:**
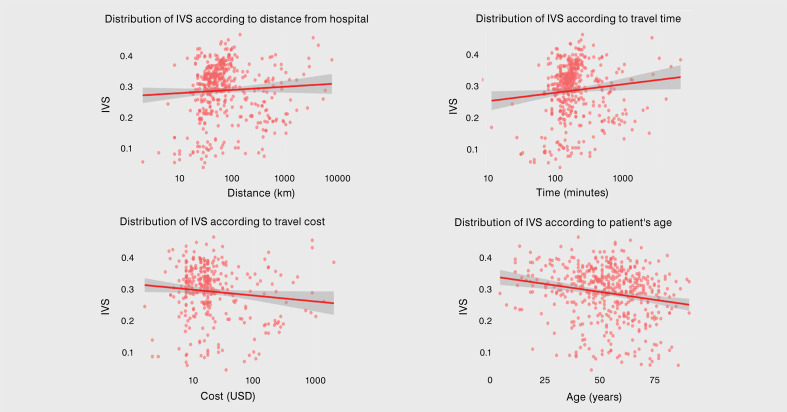
Scatter plots with regression lines illustrating the relationship between the Social Vulnerability Index (IVS) and four different variables: distance from the hospital (km), travel time (minutes), travel cost (USD), and patient’s age (years). The plots show the distribution of data points and trends, with shaded confidence intervals around the regression lines.

Most patients had low or medium IVS levels (Figure 3), which were significantly correlated with various variables (Table 3). Data analysis showed a negative correlation between IVS and age, but a positive association with the necessity to skip work, travel distance and time (Figure 4). Among patients without transportation fare exemptions, those who avoided higher travel costs had a higher vulnerability index. IVS values decreased with higher education levels, with individuals who completed higher education showing a mean IVS of 0.256, compared to 0.310 among those without formal education. Race also significantly impacted IVS: Asian and White individuals had the lowest mean IVS (0.261 and 0.275), while Indigenous, Black, and Mixed-Race individuals had higher means (0.331, 0.316, and 0.299, respectively). Male patients had a significantly higher mean IVS (0.302) than female patients (0.279), even after adjusting for age. No significant relationship was observed between IVS and public transportation use, transport gratuity, presence of an accompanying person, or device utilization.

**Table 3:** Correlation between IVS and various cohort variables.

Variable	Test	P-Value	Significance
Age	Spearman	<0.0001	****
Travel distance avoided	Spearman	0.0420	*
Travel time avoided	Spearman	0.0033	**
Expenses avoided	Spearman	0.8768	
Sex	Wilcoxon	0.0128	*
Public transport	Wilcoxon	0.1603	
Transport gratuity	Wilcoxon	0.1830	
Accompanying person	Wilcoxon	0.5822	
Own work absence	Wilcoxon	0.0420	*
Accompanying person work absence	Wilcoxon	0.9170	
Computer usage	Wilcoxon	0.3106	
Education	Kruskal-Wallis	0.0087	**
Race	Kruskal-Wallis	0.0061	**

## DISCUSSION

The results of this study provide insights into how patients’ residence location influences healthcare utilization in Brazil. The significant correlations found between IVS and both potential travel distance and time avoided highlight how social vulnerability impacts access to specialized care (Figure 3). Notably, time presented a much stronger correlation than distance, likely because it captures additional barriers beyond physical proximity, such as transportation inefficiencies, traffic conditions, and accessibility issues, which disproportionately affect socially vulnerable populations.^17^

In further sub analyses, we found that, when analyzing based on mode of transport (public versus private), correlation between IVS with both distance and time only maintained significance in the public transport group. This reflects the unique challenges they face, including limited transit routes, longer travel times due to multiple transfers, and rigid public transport schedules.^18,19^ In contrast, individuals using private transportation have greater flexibility and shorter travel times, mitigating some of the geographic barriers to care.^18,19^ This finding underscores how reliance on public transportation exacerbates the relationship between distance and social vulnerability.

Although the interquartile range for round-trip travel distances was 34–96 km, some patients needed to travel up to 7,758 km. Many require frequent follow-ups after surgical interventions but struggle to maintain appointments due to relocation or distance barriers. These findings emphasize the crucial role of telemedicine in ensuring continuity of care where in-person visits would otherwise be unfeasible.

An analysis of mean travel times in São Paulo revealed that a 39 km round-trip takes approximately 138 minutes, highlighting the inefficiencies of a transportation system reliant on buses. While not directly measured in our study, during data analysis, we observed that buses were the most accessible and widely used mode of transportation in many regions. Although the city has a railway network, its coverage remains insufficient, further reinforcing the need for expanding railway infrastructure to improve mobility and healthcare access.^20^

Regarding transportation costs, patients living outside São Paulo state faced significantly higher expenses. Even those from other cities within the state incurred an average round-trip cost of USD 31— a substantial amount in a country where the federal minimum wage is around USD 250. These findings align with studies in other Latin American countries, illustrating the widespread economic burden of travel for healthcare.^21,22^

A notable contribution of this study is the quantification of transportation fare exemptions, which benefited approximately 30% of patients. However, no significant correlation was found between fare exemptions and IVS, likely because these benefits were granted primarily based on age rather than socioeconomic status.

A significant association between IVS and the need to miss work for in-person visits was identified, indicating that socially vulnerable individuals face employment-related barriers to healthcare. Job insecurity, lack of labor protections, and inflexible workplaces may prevent timely medical visits, potentially leading to delayed diagnoses, suboptimal treatment, and worsened health outcomes.^23^ A large proportion of the Brazilian workforce is engaged in informal employment, often lacking paid medical leave.^24^ These structural issues may further increase the demand for telemedicine as an alternative to in-person care.

Interestingly, elderly telemedicine patients at our hospital were more likely to reside in areas with lower IVS. This may be explained by several factors, including greater financial stability accumulated over time, pension support, and infrastructure improvements in long-standing neighborhoods. However, selection bias may also play a role, as individuals from more advantaged areas might have better access to healthcare resources, leading to their overrepresentation in the study.^1,25^

Previous studies suggest that distance and social support influence healthcare access among older adults.^26^ When comparing patients aged ≥60 years and <60 years, we found that IVS remained significantly associated with distance and travel time only in the elderly group. This suggests that older adults are disproportionately affected by geographic barriers due to mobility limitations and comorbidities.^26^ Telemedicine serves as a valuable solution by addressing these challenges and facilitating access to care.

To further examine the role of social support, we stratified elderly patients into those with and without an accompanying person. Among those without a companion, IVS remained significantly correlated with travel distance, but this correlation was lost in those with a companion. This suggests that social support helps mitigate transportation barriers and may influence healthcare-seeking behavior.

Our study also found that 86% of patients opted to use smartphones for telemedicine consultations. Given their widespread affordability, smartphones can play a crucial role in enabling telemedicine, especially in populations with limited access to computers or broadband internet.^27,28^ However, other studies have shown that, despite high mobile penetration, digital literacy remains a barrier in vulnerable regions, emphasizing the need for inclusive policies that ensure telemedicine benefits all socioeconomic groups.^29,30^

We also identified a significant association between IVS and sex, with women exhibiting lower social vulnerability than men. This aligns with previous research showing that women seek healthcare more frequently, participate in preventive care programs, and benefit from protective social factors.^1^ These factors contribute to improved health outcomes and lower overall vulnerability. In contrast, men are more likely to underutilize healthcare services, potentially leading to delayed diagnoses and poorer health indicators.

Additionally, IVS was significantly correlated with race and education. Indigenous, Black, and mixed-race patients had higher IVS scores than White and Asian patients, likely reflecting systemic barriers such as economic inequality and limited access to resources.^1^ Similarly, lower formal education levels were associated with higher IVS, underscoring the complex interplay between education, socioeconomic status, and healthcare access. While these disparities are well-documented in Brazilian healthcare, our study highlights that they persist in the telemedicine landscape, reinforcing the need for equity-driven policies.^1^

Our study has several limitations. Transportation cost data were self-reported, which may introduce bias. Social vulnerability was estimated based on residential location, which may not fully reflect individual circumstances. Additionally, the IVS used was developed in 2010 and may not represent current socioeconomic conditions. Questionnaire responses are subject to recall and self-report biases. As a single-center study conducted in a tertiary care hospital, findings may not be generalizable to other settings or regions in Brazil. By focusing on orthopedic patients, the study does not account for the needs of other medical specialties. Selection bias is also a concern, as patients who participated in telemedicine may already have better access to technology, potentially underrepresenting the most vulnerable groups. Lastly, patients who declined participation or withdrew from the study may have different barriers to telemedicine that remain unexplored.

## CONCLUSION

This study highlights the role of telemedicine in addressing geographic and socioeconomic barriers to healthcare access among orthopedic patients in São Paulo, Brazil. By reducing travel distances, time, and costs, telemedicine improves accessibility, particularly for socially vulnerable populations who face significant mobility and financial constraints. It also demonstrates how, in Brazil, disparities in access to healthcare persist, influenced by factors such as public transportation dependency, employment-related barriers, age, sex, race and education. These findings emphasize the need for targeted policies to ensure telehealth provides equitable access to specialized care.
